# Foveated near-eye display for mixed reality using liquid crystal photonics

**DOI:** 10.1038/s41598-020-72555-w

**Published:** 2020-09-30

**Authors:** Seungjae Lee, Mengfei Wang, Gang Li, Lu Lu, Yusufu Sulai, Changwon Jang, Barry Silverstein

**Affiliations:** Facebook Reality Labs, 9845 Willows Road NE, Redmond, WA 98052 USA

**Keywords:** Optics and photonics, Applied optics, Displays

## Abstract

Foveated near-eye display is one of the most promising approaches to deliver immersive experience of mixed reality. However, it is challenged to conceive a compact optical system. Here, we introduce a method to use polarization optics via liquid crystal photonics to improve the foveated display performance. We demonstrate a benchtop prototype of this idea. We implement and combine two display modules for peripheral and foveal visions. A peripheral display consists of a polarization selective lens (PSL) module, a polarization selective diffuser (PSD), and a slanted projection system. An 80$^\circ$ diagonal field of view is achieved by on-axis optical configuration of the PSL module and the PSD. A foveal holographic display is composed of a spatial light modulator (SLM), a volume grating lens, and a microelectromechanical system mirror possibly in combination with a switchable polarization selective grating module. The holographic reconstruction using the SLM enables accurate focus cue generation and high resolution above 30 cycles per degree within 15$^\circ$ by 15$^\circ$ field of view. We explore and discuss the liquid crystal photonics in the prototype that has a novel optical design using volume gratings with polarization selectivity.

## Introduction

Near-eye display technologies for mixed reality have received wide interest as it is believed it will eventually present an unprecedented immersive experience. These technologies are anticipated to open a new large consumer market^1^ and therefore have received significant attention. The real challenge has been found to be creating both a high-quality image and a form factor that meets consumer expectations. Users desire to see high resolution images with large field of view, which refreshes at fast frame rate. Further, the headset should be comfortable to wear in daily life, which means the entire optical system must be lightweight and free from visual fatigue issues that arise by vergence-accommodation conflict (VAC)^2^. Recently, foveated near-eye displays^3^ have been introduced as a stepping-stone toward this ultimate near-eye display for immersive experience. Foveated near-eye displays deliver high resolution images along users’ eye gaze direction, along with low resolution peripheral images, which allows the display specifications to be more practical.

Despite the innovative and convincing concept of foveated near-eye displays, it is not negligible that there is a drawback that comes from a gaze steering system. One major drawback is increase in form factor by adding a steering system. In a previous work^3^ that firstly presented a proof of concept for foveated displays, motorized stages with mechanical movement were used for beam steering to follow gaze movement. The motorized linear stages were required to steer both of peripheral and foveal displays. For the peripheral display, a two-axis linear stage was used for exit pupil steering with extended eye-box. Another three-axis linear stage steered the viewing region and the depth of the foveal display to follow gaze direction and provide focus cues. Inspired by this work, we believe that the optical design space of foveated near-eye displays needs to be fully investigated and optimized for its performance, while the gaze steering systems need to be more compact and power efficient.

Here, we aim to solve the previous challenges by applying polarization optics and flat liquid crystal photonics devices. In our optical design, the gaze steering system for the foveal display consists of two components: a microelectromechanical system (MEMS) mirror, and a switchable Pancharatnam–Berry phase (S-PBP) grating module^4^. This gaze steering system is much more compact and power efficient compared to the motorized linear stage approach. Additionally, we combined the gaze steering system with the holographic near-eye displays^5^ to reconstruct focus cues in foveal region, a key to comfortable extended usage. For the peripheral displays, we also present the combination of a polarization selective diffuser (PSD) using liquid crystals and a polarization selective lens (PSL) based on folded optics. This enables the peripheral display to support enough eye-box without a pupil steering system. In this study, we demonstrate a benchtop prototype for proof of concept which enables us to evaluate foveal resolution, field of view, and focus cue reconstruction of our system. We conclude with a discussion about the limitation and future work to further improve the display performance of the foveated display.

## Results

The proposed foveated near-eye display is divided into two display modules. One is an 80$^\circ$ diagonal field of view see-through display for the peripheral vision, and the other one is a 15$^\circ \times$15$^\circ$ field of view holographic near-eye display for the foveal vision. In this section, we first introduce the wide field of view see-through display. In the subsequent section, the demonstration of the holographic near-eye display with a beam steering follows. Third, we present a benchtop prototype that combines these two display modules. The experimental results show the steering capability of the foveal display. We also evaluate the display performance in terms of image resolution, field of view, and focus cue reconstruction.

### Peripheral display: wide field of view see-through display

**Figure 1:**
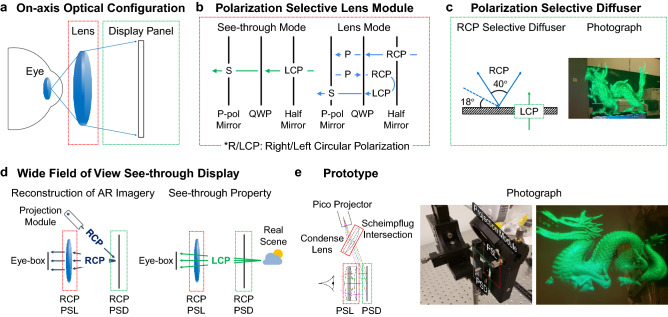
Illustration of peripheral display with wide field of view. (**a**) On-axis optical configuration that has advantages in the field of view and the eye-box. (**b**) Detailed structure of the PSL module that has two modes (lens or see-through) according to the circular polarization state. (**c**) The optical characteristics (i.e. angular and polarization selectivity) of a PSD. The PSD can be a transparent projection screen as shown in the photograph. (**d**) Proposed peripheral display combining two polarization selective optical elements: the PSL module and the PSD. Two figures describe how AR imagery and real-world scene are superimposed in the display system. (**e**) Photographs of a prototype implemented for the peripheral display. The optical aberration involved by the slanted projection is corrected by a condense lens with Scheimpflug intersection principle^6^.

For the peripheral display with the large field of view, we conceive an on-axis optical geometry with a see-through property. In the on-axis optical geometry, users wear an eye-piece lens at a close distance (15 mm) as illustrated in Fig. 1a. We note that it has a significant advantage of allowing for a wide field of view and looser tolerances for pupil movement compared to other methods using waveguide^7–9^, light guide^10^, partially reflective surface^11, 12^, or Maxwellian view structure^6, 13^. However, there are some challenges make the concept practical. First, we need to enable the eye-piece lens to be transparent for the real-world scene. Second, a transparent display module is also necessary for the see-through property. There have been similar attempts^14^ to implement an on-axis optical structure for augmented reality using an index-matched anisotropic crystal lens and a holographic optical element diffuser. Here, we present a novel display structure, which combines a PSL module and a PSD. The optical elements in our system have various advantages compared to the previous approach, which is described as follows.

The PSL module has different optical responses according to the polarization state of incident light. In Fig. 1b, for right-handed circularly polarized (RCP) light, the PSL module functions as a lens with a folding optical path which reduce the size of the lens module; for left-handed circularly polarized (LCP) light, the PSL module has no optical power. As illustrated in Fig. 1b, the PSL module consists of three layers: a partially reflective surface, a quarter-wave plate, and a linearly polarized mirror. The partially reflective surface is not polarization sensitive, which reflects 50% of the incident light with any polarization. The linearly polarized mirror is polarization selective, which transmits the s-polarized light and reflects the p-polarized light. For the lens mode, right-handed circularly polarized (RCP) incident light is converted to p-polarized light rays with the quarter-wave plate and reflected from the polarization selective mirror. The beam goes back to the quarter-wave plate, where the linear polarization is again converted to a circularly polarization of the same handedness to the initial light path, RCP. Again, the beam is reflected by the partially reflective surface, and the polarization of the beam is rotated from RCP to LCP upon reflection. Going back through the quarter-wave plate, the LCP beam is converted to the s-polarization and is able to pass the polarized mirror at this time. Finally, the light rays are ready to pass through the polarized mirror and arrive to observer, which corresponds to the lens-mode of the PSL module. In the folded optical path, the RCP light experiences the lens power three times, for instance, if the partially reflective surface has a curvature for optical power. Therefore, the effective optical power of the PSL module is tripled, with a compacted size.

Although the optical characteristic is similar to index-matched anisotropic crystal lens^14^ or PBP lens^13^, the proposed PSL module has distinct advantages. First, the index-matched anisotropic crystal lens is implemented by enveloping an anisotropic crystal lens using an isotropic material whose refractive index is matched with the extraordinary refractive index of the anisotropic crystal^14^. The index-matching materiel allows the index-matched anisotropic crystal lens to have the optical power only for the ordinary polarized light. Compared to index-matched optical lenses, the PSL module exploits a distinct optical structure that is known as a folded optics whereby the optical path is compacted into a smaller physical length (thickness of the optical elements). Inside the optical structure of this lens, there are two mirrors, one on each element so that some portion of light ray experience a round trip between the mirrors. The folded optical structure enables the effective light path length to be longer than the thickness of the optical elements. Also, folded optics usually employ concave or convex mirrors for providing optical power, which have larger numerical aperture with less chromatic aberration. Second, a conventional PBP lens reduces the size of the optics but suffers from chromatic aberration^13^. As the PBP modulation is dependent to the wavelength, the focal power of the lens varies according to colors. The chromatic aberration of a PBP lens increases with its optical power. In summary, near-eye display system using the proposed lens module could be relatively thin compared to the index-matched anisotropic crystal lens, and relatively free from optical aberration compared to the PBP lens.

To create a virtual image for see-through displays, one can combine the PSL module with a transparent display. Here, we introduce a novel optical element for a see-through projection screen, which we call polarization selective diffuser (PSD). The PSD selectively diffuses light with a specific angle, wavelength, and polarization to the normal direction. The PSD has a cholesteric structure of liquid crystal molecules^15, 16^ that forms the periodic refractive index modulation. Because of the handedness of cholesteric liquid crystals, the PSD has polarization selectivity as illustrated in Fig. 1c. As shown in the figure, the PSD is designed to be see-through for most lights (e.g., real-world scenes) expect for the projection beam with the specific polarization, angle, and wavelength. The optical response of the PSD is well matched with the PSL module that also has the polarization selectivity. Combining the PSL module and the PSD, we design the wide field of view see-through displays in Fig. 1d. Virtual images are projected to the PSD from an off-axis projector, and diffused light rays are observed through the PSL module with the integration of real-world. We implement a prototype for the peripheral display as demonstrated in Fig. 1e. The prototype allows users to observe peripheral imagery at 2 m, which is superimposed on real-world. As shown in Fig. 1e, an 80° diagonal field of view see-through display for the peripheral vision is formed with a combination of two polarization selective modules, PSD and PSL.

### Foveal display: holographic display with optical steering system

**Figure 2:**
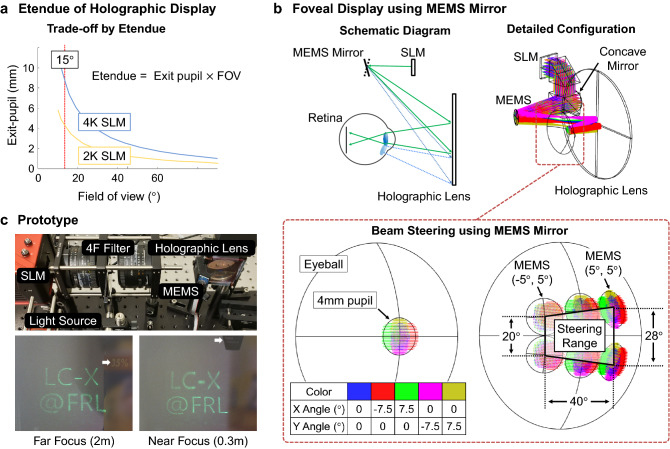
Illustration of a foveal holographic display with an optical steering system. (**a**) The trade-off between exit pupil and field of view in holographic displays, which is not an issue in narrow field of view system. (**b**) Schematic description of foveal display using a MEMS mirror to steer angular position of viewing window. Detailed optical configuration for a prototype is supported by Zemax simulation. (**c**) Implementation of a prototype for the foveal holographic display. Focus cue reconstruction is demonstrated by experimental results. Due to copy right permission, we blurred portion of the image.

For the foveal vision with the high resolution to match the human vision limit (30 cycles per degree, cpd), we adopt a holographic near-eye display^5^ among several candidates, with these following reasons. First, we note that field of view for foveal displays is not necessary to be wide. Due to etendue constrains, there is a trade-off between eye-box and field of view^17^ in holographic displays, as illustrated in Fig. 2a. If the peripheral display supports the resolution of 5 cpd, however, a 15$$^\circ$$$$\times$$ 15$$^\circ$$ field of view is necessary for the foveal display to surpass the human visual acuity. Second, it is feasible to enable a compact optical system^5^ with a holographic display as shown in Fig. 2b. As demonstrated in the previous research^5, 17^, the holographic display can also correct the optical aberration that comes from the holographic optical element. The capability for aberration correction is important for steering of foveal display where the beam steering causes additional optical aberration. Third, holographic displays can provide focus cues, which is important for immersive mixed reality experience^2, 18, 19^. The capability of focus cue reconstruction is demonstrated by experimental results shown in Fig. 2c. As we can see in the results, the two lines of letters are floated at different distance from the camera. Fourth, holographic displays can adjust high resolution area for the foveated display application^20^ exploiting appropriate computer-generated holograms.

As illustrated in Fig. 2b, the proposed foveal display system consists of a SLM, a MEMS mirror, and a holographic lens. The MEMS mirror is located between a concave mirror and a holographic lens. Note that the concave mirror converges the beam reflected from the SLM so that the small aperture size (5 mm) of the MEMS mirror is enough to steer incident beam. Then, the incident beam is reflected by the MEMS mirror and introduced to the holographic lens. Finally, a user observes holographic images formed by the reflectively diffracted beam from the holographic lens. This optical design has contributions compared to the related works of foveated near-eye displays^3^ and holographic near-eye displays^5, 17^. First, the optical steering system using the MEMS mirror substitutes for the motorized linear stages used in the previous foveated near-eye displays^3^. The MEMS mirror enables an optical design with more compact form factor, lower power consumption, and faster steering speeds. Second, the holographic lens converges the incident beam from the MEMS mirror into the central point of the eyeball. The eyeball centric design allows the MEMS mirror to modulate the angular position of the viewing window without additional beam steering holographic optical element^17^. If the steering angle of the MEMS mirror changes, the steered beam is introduced to a different region of the holographic lens. The diffraction region of the holographic lens determines the angular position of holographic images as illustrated in Fig. 2b. In this configuration, we could steer the viewing window up to 40$^\circ \times$20$^\circ$ using a MEMS mirror with $\pm$ 5$^\circ$ steering range. Also, the optical design can support instantaneous field of view of 15$^\circ \times$15$^\circ$ with the exit pupil larger than 1.9 mm. According to the Abbe diffraction limit and Wigner distribution function^17^, the specifications secure 30 cpd vertical resolution in foveal region, which can surpass visual acuity with 5 cpd peripheral displays.

**Figure 3:**
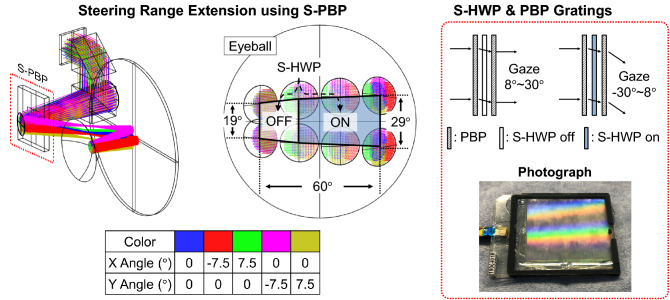
The optical principle of the steering range extension using a S-PBP module. The proposed method is verified by Zemax simulation. In-house fabricated S-PBP’s photograph is also presented on the right-hand side. Note that the rainbow in the photograph is the grating effect of the PBP grating.

To extend the steering range of the foveal display with existing MEMS mirror capability, we endorse the concept of switchable Pancharatnam–Berry phase (S-PBP) optical elements^21–23^ with a compacted size. In a related work presented by Zhan et al.^22^, the S-PBP was exploited for discrete beam steering of foveal display. In our work, we developed a continuous beam steering by combining the MEMS mirror and the S-PBP module. The MEMS mirror enables continuous beam steering while the S-PBP module extends the beam steering range approximately twice. As illustrated in Fig. 3, the S-PBP module has three layers: a switchable half wave plate (S-HWP) between two identical passive PBP gratings. Rainbow shown in Fig. 3 is a proof of the grating effect of the PBP grating. Note that the polarization state of the incident wave is converted to the orthogonal state after passing through the PBP grating. In our design, the diffraction angle of a single PBP grating is 7.5$^\circ$ with > 99% diffraction efficiency. The S-HWP is a liquid crystal element whose retardation is electronically modulated between a half and a full waveplate, and it rotates the handedness of circularly polarized light. When the S-HWP functions as a half wave plate, the diffraction angles of the PBP grating pair are cancelled. On the other hand, the diffraction angle is doubled when the S-HWP functions as a full wave plate. Shortly, the S-HWP module allows us to turn on or off the function of the PBP grating by the electric modulation of the supplied voltage.

In our design, we divide the angular position of viewing window into two parts. One is for the on-state of the PBP grating, and the other is for the off-state. Pairing each state with the steering angle of the MEMS mirror, we can design an optical system with larger steering range of the viewing window. The switching speed between two states should be fast enough to ensure that users may not recognize the discrete steering movement. The refresh time of the S-HWP should be at least less than 1/60 s according to the frame rate of the human vision. At the same time, the MEMS should finish the optical steering to follow the state switching of the S-HWP. Note that we demonstrate the foveal display with the S-PBP module by Zemax simulation shown in Fig. 3. The S-PBP grating module resolves the limitation of a MEMS mirror caused by the trade-off between its aperture size and steering speed. As of today, a commercially available state-of-the-art MEMS mirror gives the $\pm$ 20$^\circ$ horizontal steering range of the viewing window. To secure high numerical aperture and fast refresh rate, this specification allows us to implement a foveal display with dynamic field of view of 55$^\circ$ (40$^\circ$ dynamic and 15$^\circ$ instantaneous field of view). By adding the S-PBP grating module, the dynamic field of view of the foveal display can be extended to 75$^\circ$ with the off-the-shelf MEMS mirror.

### Evaluation and analysis of foveated near-eye displays

#### Experiment

**Figure 4:**
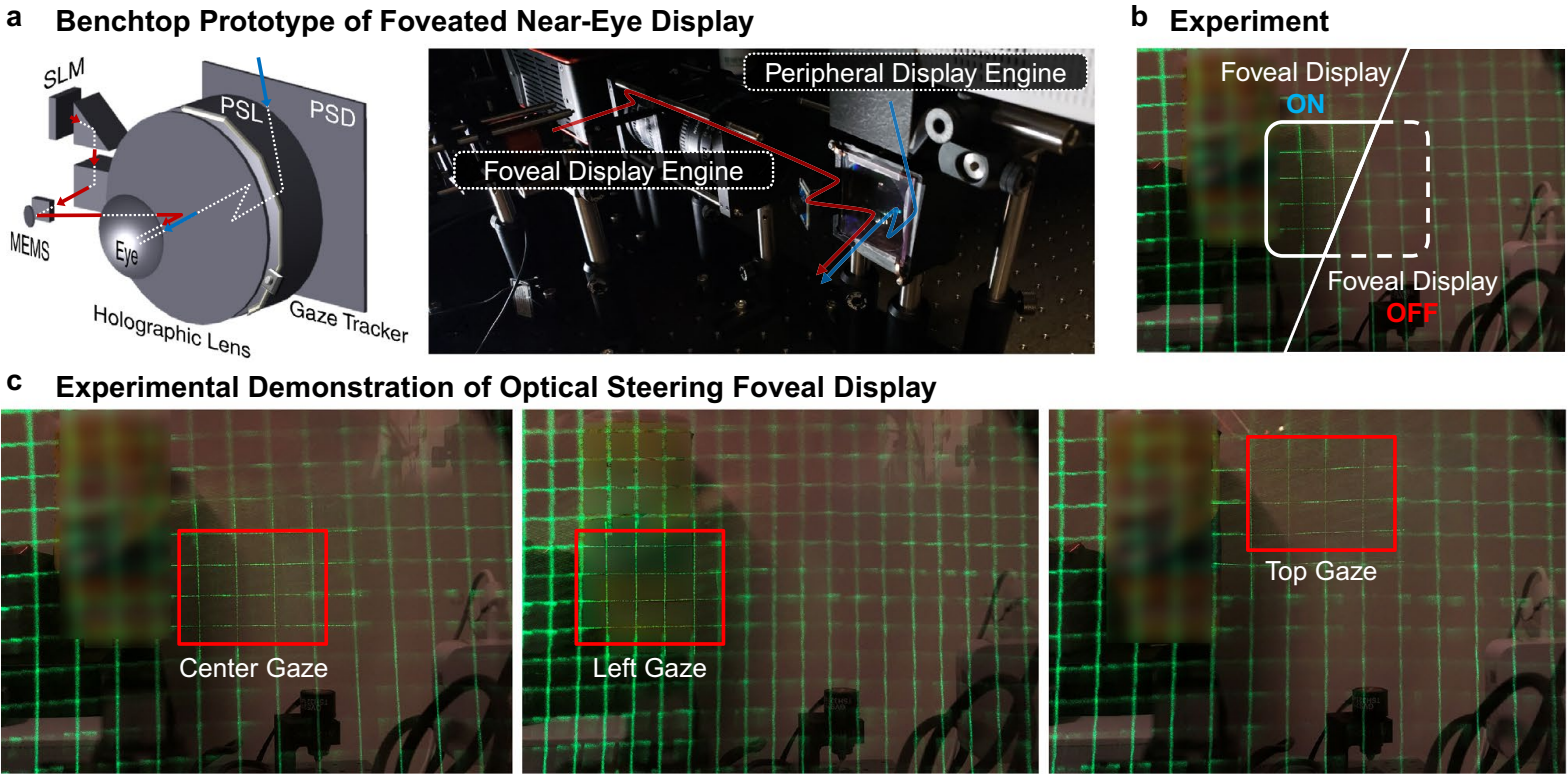
Demonstration and implementation of the foveated near-eye display. (**a**) Schematic diagram and a photograph of the benchtop prototype. (**b**) Experimental result to show the resolution enhancement in the foveal region. We compare two experimental results where the foveal display is on or off. (**c**) Demonstration of optical steering the viewing window of the foveal display using the MEMS mirror. Optical steering results for horizontal and vertical direction are presented. Note that the resolution enhancement by the foveal display is more significant than the demonstration in these photographs. The photographs are captured by a mobile phone camera where the maximum angle resolution is less than the resolution limit of the foveal display (30 cpd). Due to copy right permission, we blurred portion of the image.

Figure 4 shows the integrated prototype that combines the peripheral and foveal images. A grid image is displayed to demonstrate the foveated near-eye display experience. As demonstrated in the figure, the grid lines at the gaze direction has higher definition compared to the peripheral region (confirmed through measurement in the next section). The foveal image using the MEMS mirror has $\pm$ 20$^\circ$ horizontal steering range and $\pm$ 10$^\circ$ vertical steering range. The specifications are verified by Zemax simulation and demonstrated experimentally. Further, the steering range of the foveal display can be extended by optimizing the choice of MEMS mirror and S-PBP grating module. Although experimental demonstration of this approach is not presented, the feasibility and its advantages are verified by Zemax simulation. To note, combining with a gaze tracking system, the foveal display can follow the gaze direction, which is not included here.

#### Field of view and eye-box

**Figure 5:**
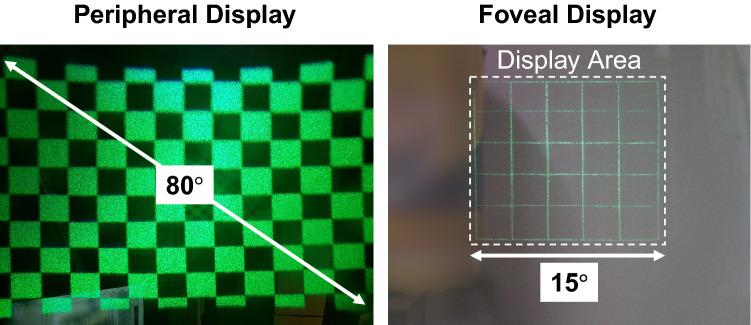
Field of view evaluation of the implemented peripheral and foveal displays.

Field of view is one of the most important specification considered for the optical designs of peripheral and foveal displays. Zemax software was used to determine detailed specifications including distanced between optical elements, focal power of lenses, and incident angle of projection system. To verify the optical design, we experimentally measured the field of view of peripheral and foveal displays by capturing a checkboard or a grid image. For the peripheral display, the diagonal field of view is 80$$^\circ$$; the foveal field of view is 15$$^\circ$$$$\times$$ 15$$^\circ$$ (diagonal field of view 21$$^\circ$$). Figure 5 demonstrates the captured images used for field of view estimation. In this configuration, the eye-box of the peripheral display is 10 mm and the instantaneous exit pupil of the foveal display is about 2 mm. Note that the exit pupil of the foveal display is steered by the MEMS mirror according to the gaze direction. As the average steering range of the prototype is $$\pm$$ 15$$^\circ$$, the lateral location of the exit pupil is shifted up to $$\pm$$ 3.35 mm. Thus, the dynamic eye-box of the foveal display is 8.7 mm.

#### Resolution

**Figure 6:**
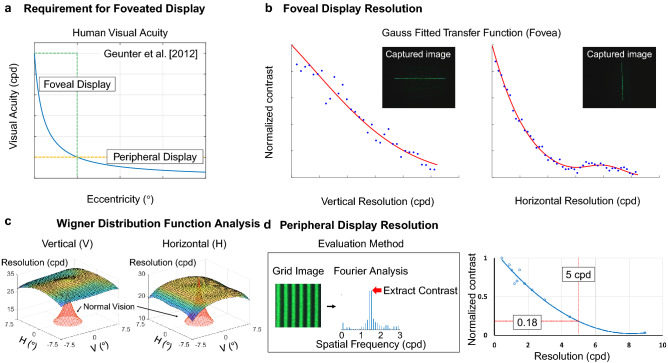
Resolution analysis and evaluation of the foveated near-eye display. (**a**) Requirement of foveated display to meet the resolution limit of human visual acuity. (**b**) Experimental evaluation of resolution limit of the foveal display. (**c**) The resolution limit of the foveal display is analyzed and compared with the human visual acuity using Wigner distribution function^17^. (**d**) Experimental evaluation of resolution limit of the peripheral display.

Figure 6 illustrates the resolution limit of human visual system according to the eccentricity, which is the angular distance from the fovea. The resolution limit at the fovea is estimated by perceptual studies^24^, which is around 30 cycles per degrees (cpd). On the other hand, the visual acuity is decreased at the peripheral region beyond. The resolution limit is known to be approximately proportional to a reciprocal number of the eccentricity^24^. Using the trade line of the human visual acuity, we can determine the requisites of the foveal and the peripheral displays. First, the foveal display should provide 30 cpd resolution at the fovea. Second, the resolution of the peripheral display is determined by the field of view of the foveal display. For instance, when the foveal display supports the instantaneous field of view of 15$^\circ$, peripheral display’s resolution should outperform the resolution specification at the eccentricity of 7.5$^\circ$. Accordingly, the resolution requirement of the peripheral display is 5 cpd.

To evaluate the performance of the foveal display, a vertical or horizontal line is reconstructed and captured by a CCD camera, as shown in Fig. 6b. We note that Fourier transform of the captured image is the Fourier transform of line spread function. Thus, the Fourier transform of the captured image is a reference to evaluate the transfer function of the foveal display system. As demonstrated in Fig. 6b, we confirm that the foveal holographic display supports 30 cpd resolution especially for vertical resolution. The horizontal resolution is less than the vertical resolution because of the oblique illumination. The horizontal exit-pupil size is sacrificed by the oblique illumination, which degrades the horizontal resolution limit.

Figure 6b,c illustrates the resolution limit analysis of the foveal holographic display according to the eccentricity. In Fig. 6c, we compare the foveal resolution with the normal vision’s acuity. The horizontal resolution is a little insufficient to surpass the normal vision’s acuity at the fovea. However, the insufficient area is less than 0.53$^\circ$ eccentricity, which is only 3.5% of the foveal display’s viewing window. Also, this little deficit of the horizontal resolution could be compensated by demagnification optics for the SLM. A horizontally demagnified SLM can support higher numerical aperture, which can extend the exit-pupil size. For the vertical axis, the foveal display reconstructs higher resolution image than human visual system’s resolution limit.

The resolution of peripheral displays is evaluated using Fourier transform of a grid image. The interval of each square is modulated according to the spatial frequency that we want to measure. As described in Fig. 5d, the grid image for a specific spatial frequency is displayed and captured by camera. The Fourier transform of the captured image is used to calculate the contrast of the corresponding spatial frequency. The contrast is a reference to evaluate the transfer function of the optical system. The transfer function is estimated by gathering the absolute values at the specific spatial frequency range in the Fourier domain. The contrast curve is normalized by the contrast of the grid image with low frequency. As shown in Fig. 5d, the contrast ratio of the peripheral displays is ~ 0.18 while the image resolution is 5 cpd.

#### Limitation and future work

In Table 1, we summarize the competence of our prototype compared to other foveated display prototype introduced by Kim et al.^3^. In our design, the foveated display is enabled without any motorized stages, which is the leading advantage. In our design, the on-axis peripheral display is based on polarization selective optics, and the optical steering design combines the strengths of a MEMS mirror and the S-PBP grating module. In addition, the foveal focus cue is reconstructed by holographic displays while previous prototype^3^ employs the varifocal method. Holographic displays allow display system to reconstruct volumetric scene with multiple focal depths simultaneously rather than tracking the focal depth of users.

**Table 1:** Comparison of our prototype with the related work presented by Kim et al.^3^.

Display type	Kim et al.’s Prototype (IC3)		Our prototype	
	Fovea	Periphery	Fovea	Periphery
	Birdbath	Retinal projection	Holographic display	On-axis
Transparency	Half mirror	HOE	HOE	Polarization selectivity
Focus cue	Varifocal	No	Holographic	No
	1D linear stage			
Gaze steering	Required	Required	Required	Not required
	2D linear stages	2D linear stages	MEMS	
	205°/s	1053°/s	2400°/s	
	± 20° (horizontal)	12 mm (horizontal)	± 15° (horizontal)	
Resolution	60 cpd	4 cpd	30 cpd	5 cpd
Horizontal FOV	16°	81° (at center)	15°	70°

Despite the advantages described in the table, our prototype still has some limitations and requires further researches to improve the display performance. First, a prototype with full color expression is not demonstrated in this work. For full color demonstration, it is necessary to fabricate a PSD with broadband wavelength selectivity. To implement the full color PSD, we can simply stack three different PSDs for each color channel. A full color holographic lens is also necessary for the full color foveal display. Several methods such as wavelength multiplexing can be adopted for the full color expression of the holographic lens. Second, the optical aberration of the slanted projection system for the peripheral display was not fully corrected. Although we designed a primitive aberration correction optics inspired by the Scheimpflug intersection principle, we could observe severe optical aberration especially for the larger angular view. Compact optical design for the aberration correction could be an interesting research topic.

Third, the prototype has low light efficiency because of the half mirror in the PSL module. The peripheral display’s light efficiency is at the most 25% because light rays for virtual imagery encounter the half mirror twice. The transparency of the system is also limited to 25% because real-world light passes through the linearly polarized mirror and the half mirror. Fourth, the steering range extension using the S-PBP was only demonstrated by Zemax simulation, and the experimental proof of concept is necessary. We can also implement foveal displays with the 2D steering range extension using two S-PBP gratings each of which extends the steering range for the vertical and horizontal directions.

The reliability of gaze steering system is also an important bottleneck of foveated display system. The most conservative specification of the foveal display’s steering speed is 500°/s^26^, which is known as the fastest gaze movement referred to as a saccade. To follow the saccade, the MEMS mirror speed should be above 250°/s and 125°/s for the vertical and horizontal directions. The MEMS mirror (A8L2.2) supports 120 Hz step response for both axis, which corresponds to 1200°/s. Also, the positional repeatability of the MEMS mirror is reported as better than 0.001°, which is negligible. With the MEMS steering, the hologram on the SLM should be refreshed to display appropriate foveal image. If users gaze at 15° direction, the foveal display should show 15° location image to maintain alignment of foveal and peripheral images. The best scenario is to synchronize the foveal image with the MEMS mirror steering angle. To ensure that observers would not recognize unexpected artifacts such as flickering effect, the foveal display should be refreshed at 60 Hz. However, real-time hologram rendering could be challenging in this kind of holographic near-eye display system^5, 17^. Hologram rendering should consider the variation of foveal image’s depth and HOE optical aberration according to the gaze direction.

Foveated displays could be vulnerable to errors in gaze tracking, because the exit pupil could be steered to an inappropriate position. When the eye’s pupil size is 4 mm, users can miss foveal images if the exit-pupil shift error exceeds 2 mm. This error corresponds to 4.6° gaze tracking error, where eyeball’s diameter is 25 mm. From a more conservative aspect, at least 2.67 mm exit-pupil of foveal images should be retained to achieve 30 cpd resolution. In this case, 0.67 mm exit-pupil shift error is tolerable, which corresponds to the gaze tracking error of 1.6°. State-of-the-art gaze trackers from Pupil-labs have an accuracy of 0.6°, which estimate the gaze direction by capturing eye images at 200 Hz. In Patney et al.’s work^27^, a desktop foveated display used a gaze tracker with an accuracy of under 1° and a response latency of 5 ms. The central foveal radius for this setup was 7.5°, which is identical with our foveated display’s FOV. According to the results of perceptual experiments, the gaze tracker’s specification was enough to provide foveated viewing experience without noticeable artifacts.

## Discussion

In this study, we introduced the foveated near-eye display combining two display systems. The foveated near-eye display consists of the peripheral display with wide field of view and the foveal holographic display with high resolution. We implemented a benchtop prototype to verify the feasibility of the proposed display. The prototype supports the 80$^\circ$ peripheral field of view with the 15$^\circ$ foveal field of view. By experimental analysis, we evaluated the peripheral and foveal resolution as 5 cpd and 30 cpd respectively. These specifications allow the resolution of AR imagery to surpass the resolution limit of the human visual acuity. Also, focus cues were reconstructed in the foveal region so that observers can focus on the interested objects. We expect that the foveal focus cue reconstruction can alleviate the vergence accommodation conflict. For the implementation of the prototype, we introduced novel optical elements including the PSL, the PSD, and the S-PBP grating. The polarization selective optical elements contributed to thickness reduction and brightness improvement of the peripheral display. In the foveated display, the S-PBP grating module is a key element to resolve the limited steering range of the MEMS mirror. We evaluated and analyzed the display performance of the foveated near-eye display via Zemax simulation as well as experimental demonstration. Also, the optical characteristics of the PSD were thoroughly analyzed in terms of the polarization, wavelength, and angle selectivity. We believe that the novel optical elements would inspire further enhanced display systems for mixed reality.

## Methods

### Wigner distribution function for resolution analysis

In Fig. 6c, we presented the Wigner distribution function (WDF) analysis^17^ to verify the experimental result. The WDF is an optical field defined on the spatial and angular domain, which can represent the resolution limit of holographic displays^25^. The spatial boundary of the optical field denotes the exit-pupil size, while the angular component of the optical field corresponds to the eccentricity. We calculate the WDF on the pupil plane by Zemax, and estimate the maximum frequency of reconstructed image. The maximum frequency $\theta$ is given by $\theta = \tan^{ - 1} \left( {\lambda /2nD} \right)$, where $\lambda$ is the wavelength, $n$ is the refractive index, and $D$ is the exit-pupil size. This equation predicts the resolution limit of holographic displays according to the Abbe diffraction limit, assuming a 532 nm illumination and 1.376 refractive index of the eye.

### Fabrication of polarization selective diffuser (PSD)

The fabrication process of the PSD is divided into three steps. The first step is to record an interference pattern of a coherent light source on a photoalignment material. Compared to the recording process of holographic optical elements, two circularly polarized waves with opposite states are interfered on the sample plane. The interference of cross circularly polarized waves is employed to formulate a specific distribution of linear polarization direction of the illumination on the sample illumination.

The PSD used in this study was fabricated using an interference pattern between cross circularly polarized lights with the incident angle difference of 25.2$^\circ$. The angle difference allows the diffuser to have a volume grating structure that diffracts illumination with 72$^\circ$ incident angle to the normal direction. For diffusing characteristics, we employed the interference between a plane wave and a diffusing wave passed through a holographic diffuser with 40$^\circ$ diffusing angle.

If the photoalignment material is exposed to the interference pattern, the polarization direction of molecules is aligned to be orthogonal to the linearly polarized illumination. Note that the wavelength of the coherent light source is determined by the absorption characteristic of the photoalignment material. Absorption coefficients of the photoalignment materials are usually below 450 nm or ultra-violet wavelength region. We used a 455 nm laser for the PSD fabrication.

**Figure 7:**
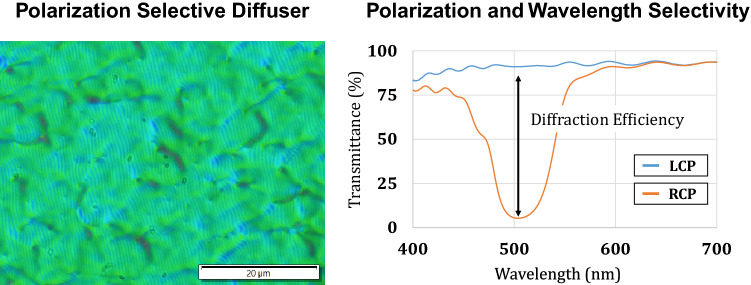
Optical characteristics of the fabricated PSD. We present a microscope image of the PSD, which demonstrates the randomized grating property that gives diffusive optical response to the incident light. The transmittance according to the polarization and the wavelength is measured to evaluate the selectivity given by the volume grating structure.

Second, cholesteric liquid crystal is developed on the photoalignment material via spin coating. The development material determines the period of cholesteric structure of liquid crystal molecules, which is an important term for the wavelength selectivity. In this study, we fabricated the PSD optimized for 532 nm (green light). Final procedure for the PSD fabrication is a curing process using UV lamps. The optical characteristics of the fabricated PSD is demonstrated in Fig. 7.

### Implementation

For the peripheral display, we used a pico laser projector with a condensing lens with a focal length of 25 mm. The condense lens was tilted by 42$^\circ$ from the optical axis of the pico projector. The distance between the condense lens and the exit-pupil of the pico projection was 35 mm. The center of the PSD is separated by 62.7 mm from the condense lens. All detailed variables for this optical design were optimized by Zemax software with consideration of matrix spot diagram on the PSD. The angle of incidence to the PSD is 72$^\circ$ from the pico projector. The PSL module is separated by 18 mm from the PSD so that virtual imagery is floated at the 2 m. The optical mounts for these optical elements were manufactured by a 3D printer.

For the foveal holographic display, we used a 4 K SLM panel from Thorlabs (EXULUS-4K1) for holographic display. The surface reflection noise from the 4 K SLM panel was removed by a 4F filtering system using two camera lenses (Nikko 50 mm f/1.8D). The 4F plane of the SLM was separated by 2 mm from the first beam splitter. The two beam splitters (BK7) have 10 mm thickness and they are separated by 5 mm. The distance between the second beam splitter and the concave lens is 1 mm. The focal length of the concave mirror is 50 mm. The MEMS mirror from Mirrocle (A8L2.2) was located at 36 mm distance from the concave mirror. The mirror reflects the converging wave to the holographic lens.

The distance between the MEMS mirror and the center of the holographic lens is 36.5 mm. The angle of incidence to the holographic lens is 50$^\circ$. For this configuration, the holographic lens was fabricated by using two spherical waves of the green laser (wavelength 532 nm). One has the 50$^\circ$ incident angle from the 36.5 mm distance, and the other one has the 0$^\circ$ incident angle from the 27.5 mm distance. We assume that the eye-relief is 15 mm and the eyeball radius is 12.5 mm.

### Calibration of slated projection for peripheral display

We calibrate the slanted projection for the peripheral display to correct the image distortion. Without the calibration process, the peripheral display has severe image distortion To find the system matrix for distortion correction, we first display a checkerboard as a target image. The CCD camera captures the checkerboard and find the four edge points, and first correct the perspective distortion (**S**_1_). The system matrix was calibrated by 5 iterations. Second, the curved horizon distortion was corrected. The system matrix **S**_2_ for this distortion was found by 1 iteration. After the calibration, we can derive pre-distorted images by multiplying the inverse matrix of the entire system matrix given by ${\mathbf{S}}_{2}^{ - 1} {\mathbf{S}}_{1}^{ - 1}$.

## Data Availability

The data supporting the figures in this paper and other finding of this study are available from the corresponding author upon request.
